# Supplementary material for: Genomic alterations and abnormal expression of APE2 in multiple cancers
Source: Sci Rep. 2020 Feb 28;10:3758. doi: 10.1038/s41598-020-60656-5 (PMC7048847; doi:10.1038/s41598-020-60656-5)
Supplement: Supplementary file 1 — Supplementary Information. [file 41598_2020_60656_MOESM1_ESM.pdf]

Genomic alterations and abnormal expression of APE2 in multiple cancers

Katherine A. Jensen[1,2], Xinghua Shi[2,3*], Shan Yan[1*]

[1] Department of Biological Sciences, University of North Carolina at Charlotte, 9201 University City Blvd., Charlotte, NC 28223, USA

[2] Department of Bioinformatics and Genomics, University of North Carolina at Charlotte, 9201 University City Blvd., Charlotte, NC 28223, USA

[3] Current Address: Department of Computer & Information Sciences, Temple University, 1925 N. 12th Street, Philadelphia, PA 19122, USA

*email: shan.yan@uncc.edu; mindyshi@temple.edu

Supplementary Figures and Legend

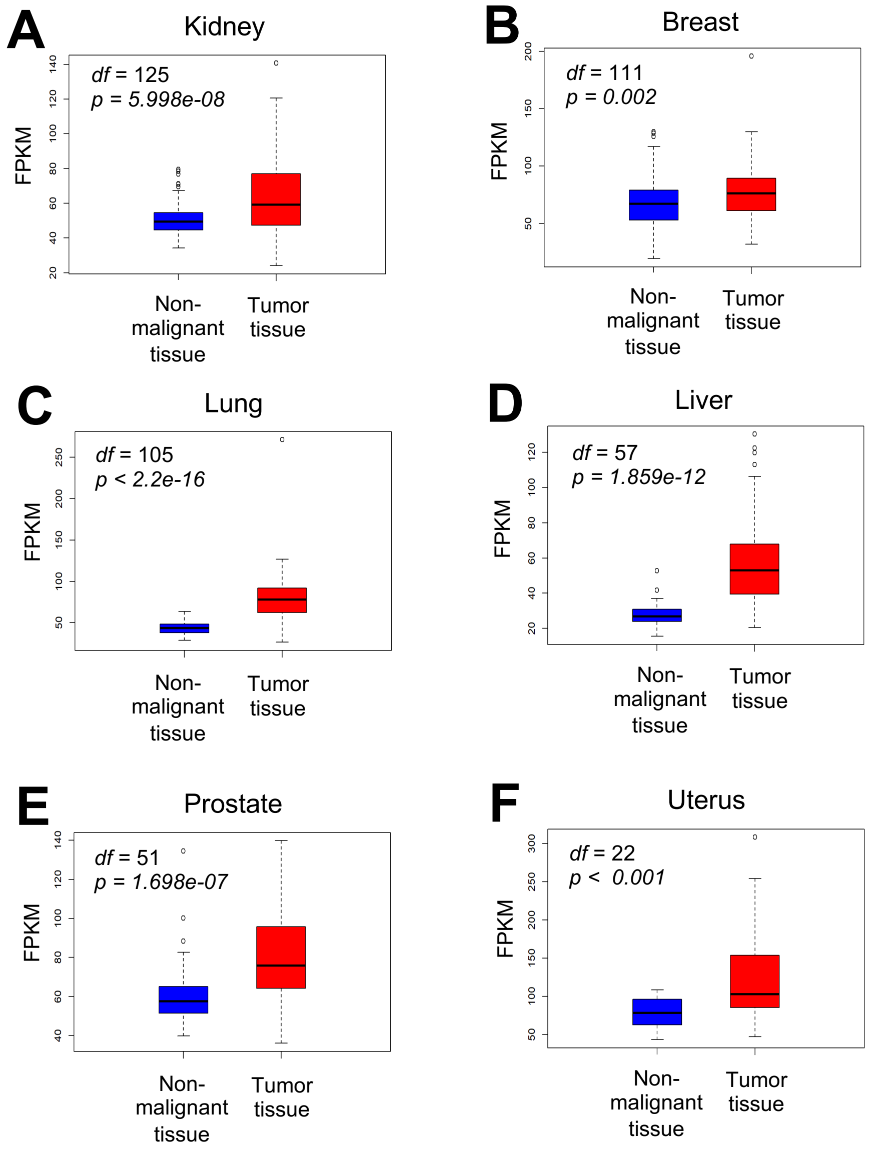

Fig. S1. APE1 mRNA expression between tumor tissue and matched non-malignant tissue per individual from 6 different cancer types including kidney (**A**), breast (**B**), lung (**C**), liver (**D**), prostate (**E**), and uterus (**F**).

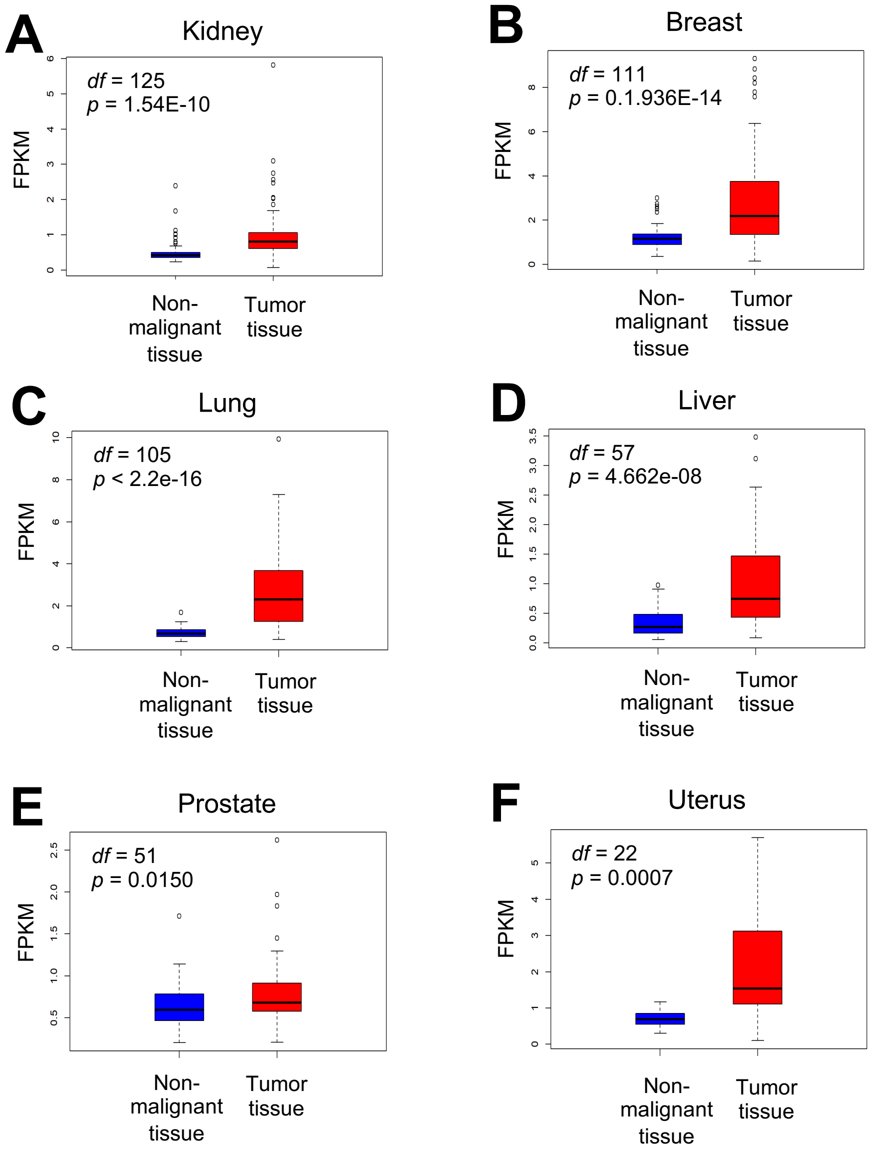

Fig. S2. BRCA1 mRNA expression between tumor tissue and matched non-malignant tissue per individual from 6 different cancer types including kidney (**A**), breast (**B**), lung (**C**), liver (**D**), prostate (**E**), and uterus (**F**).

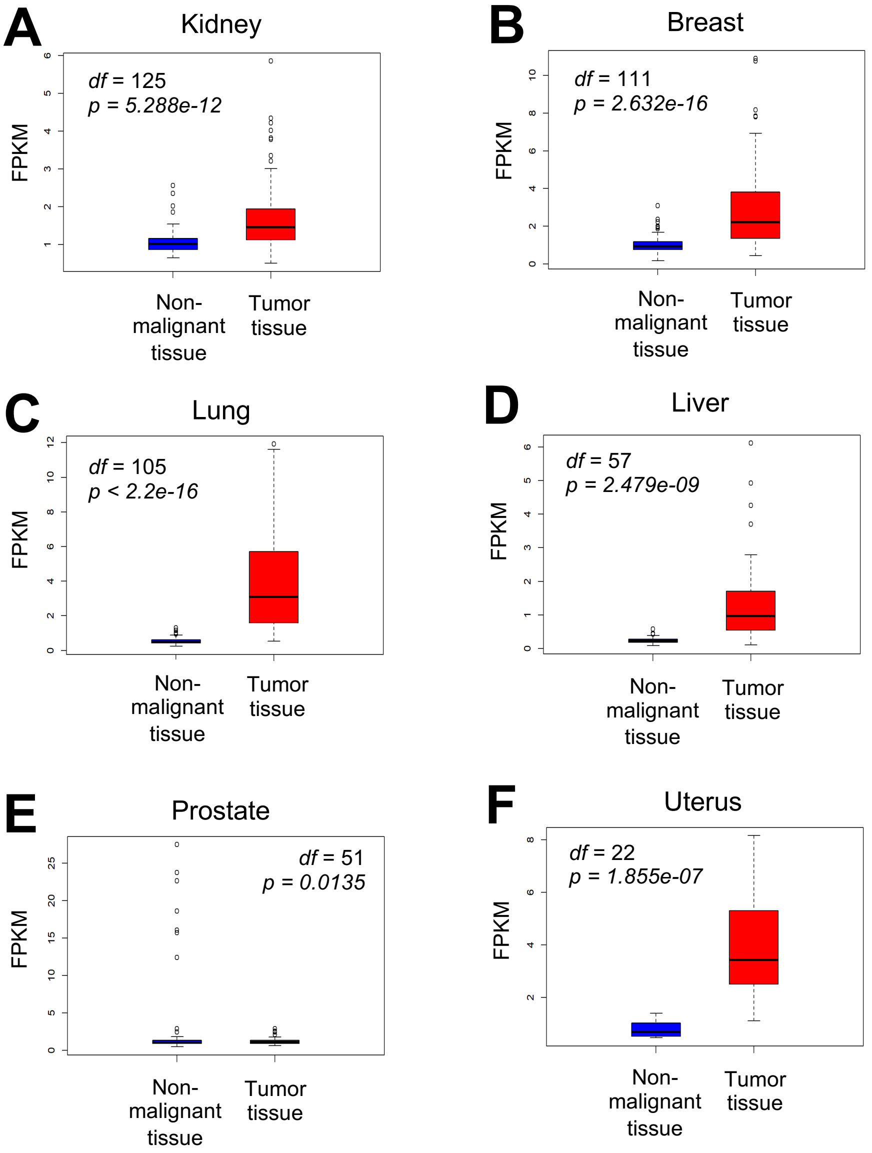

Fig. S3. Chk1 mRNA expression between tumor tissue and matched non-malignant tissue per individual from 6 different cancer types including kidney (**A**), breast (**B**), lung (**C**), liver (**D**), prostate (**E**), and uterus (**F**).

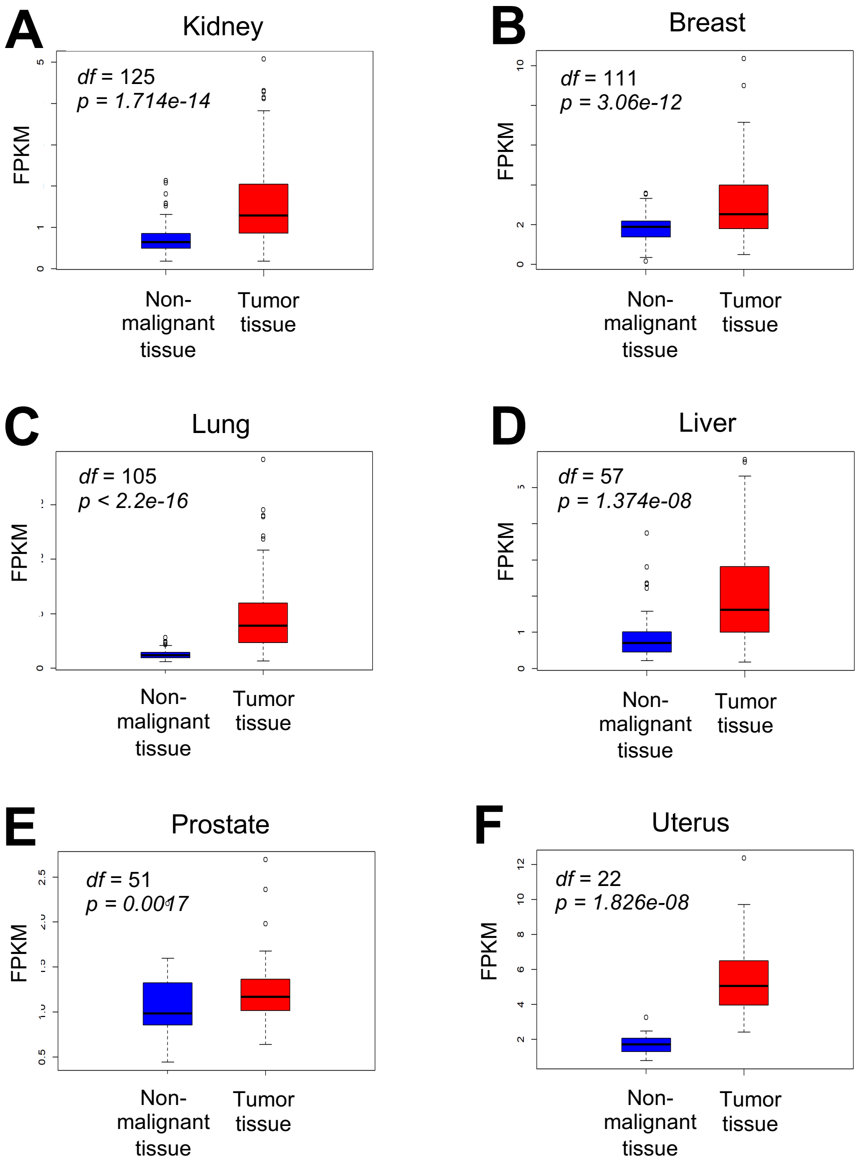

Fig. S4. Chk2 mRNA expression between tumor tissue and matched non-malignant tissue per individual from 6 different cancer types including kidney (**A**), breast (**B**), lung (**C**), liver (**D**), prostate (**E**), and uterus (**F**).

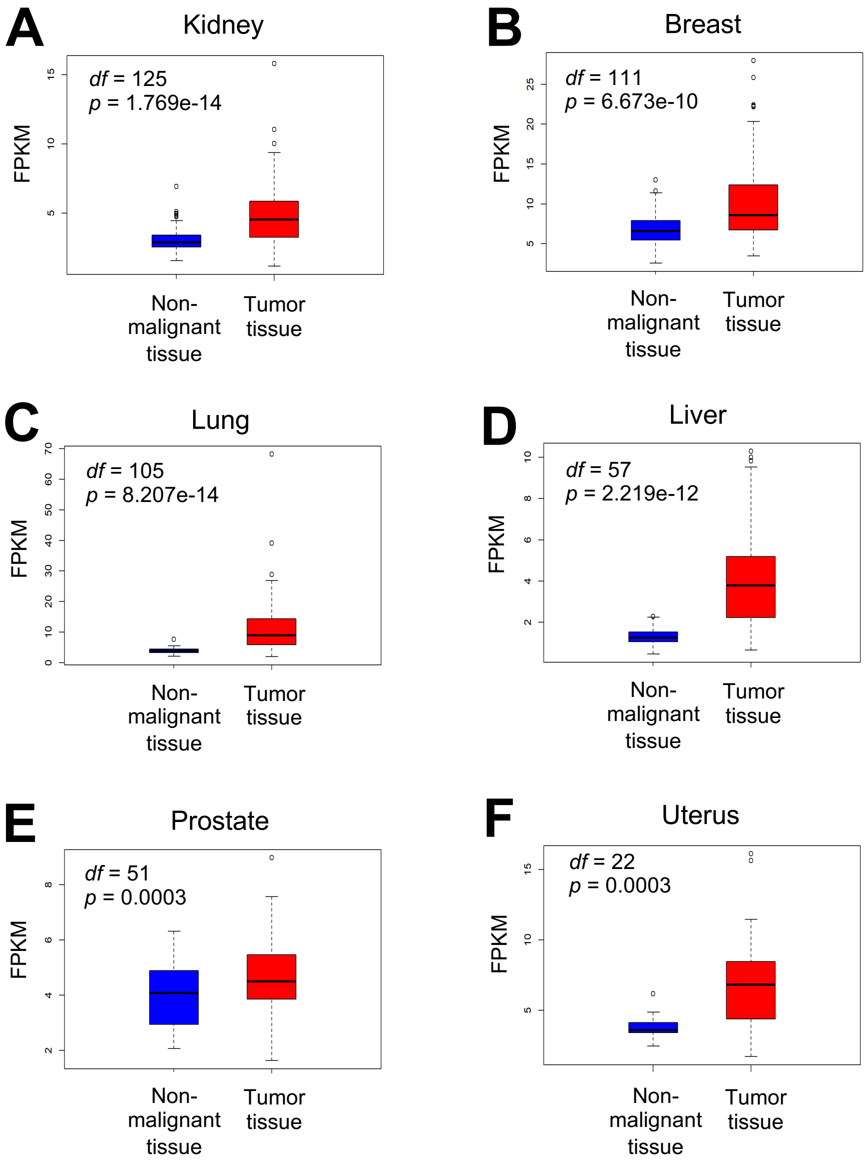

Fig. S5. TopBP1 mRNA expression between tumor tissue and matched non-malignant tissue per individual from 6 different cancer types including kidney (**A**), breast (**B**), lung (**C**), liver (**D**), prostate (**E**), and uterus (**F**).

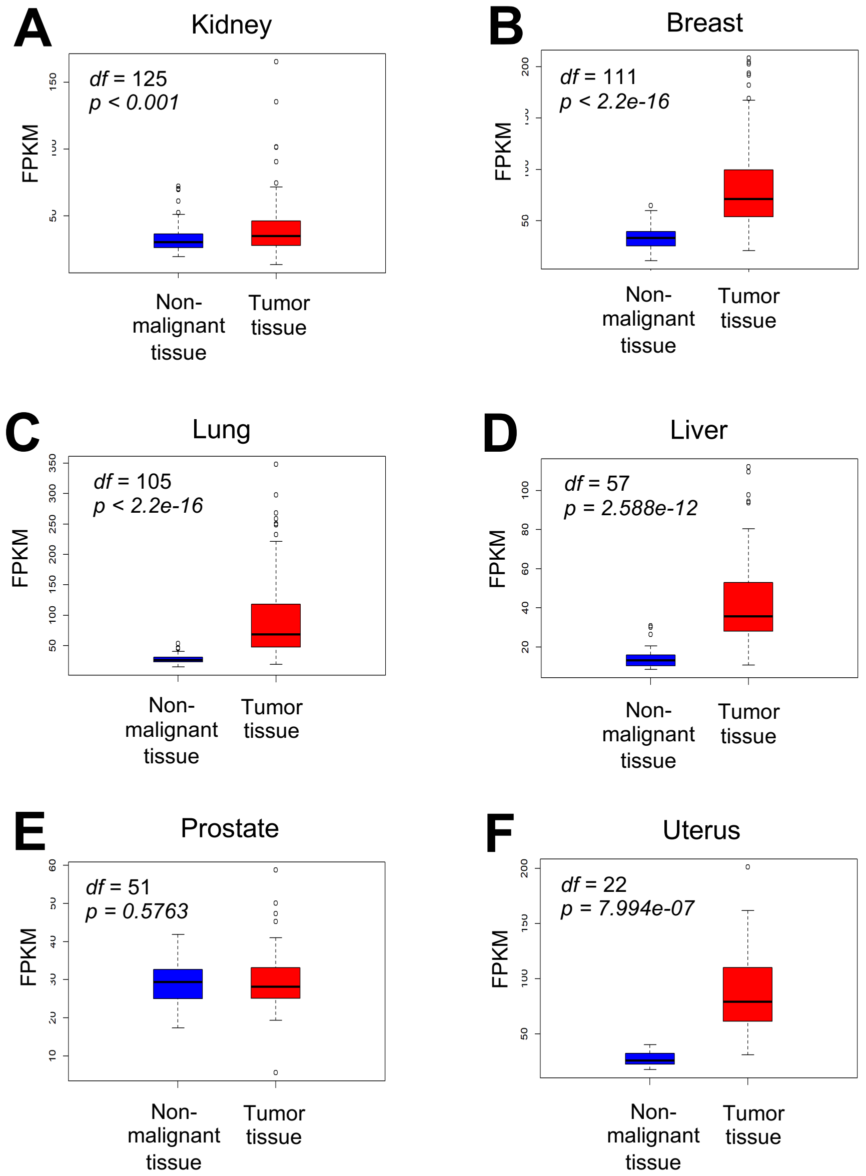

Fig. S6. PCNA mRNA expression between tumor tissue and matched non-malignant tissue per individual from 6 different cancer types including kidney (**A**), breast (**B**), lung (**C**), liver (**D**), prostate (**E**), and uterus (**F**).

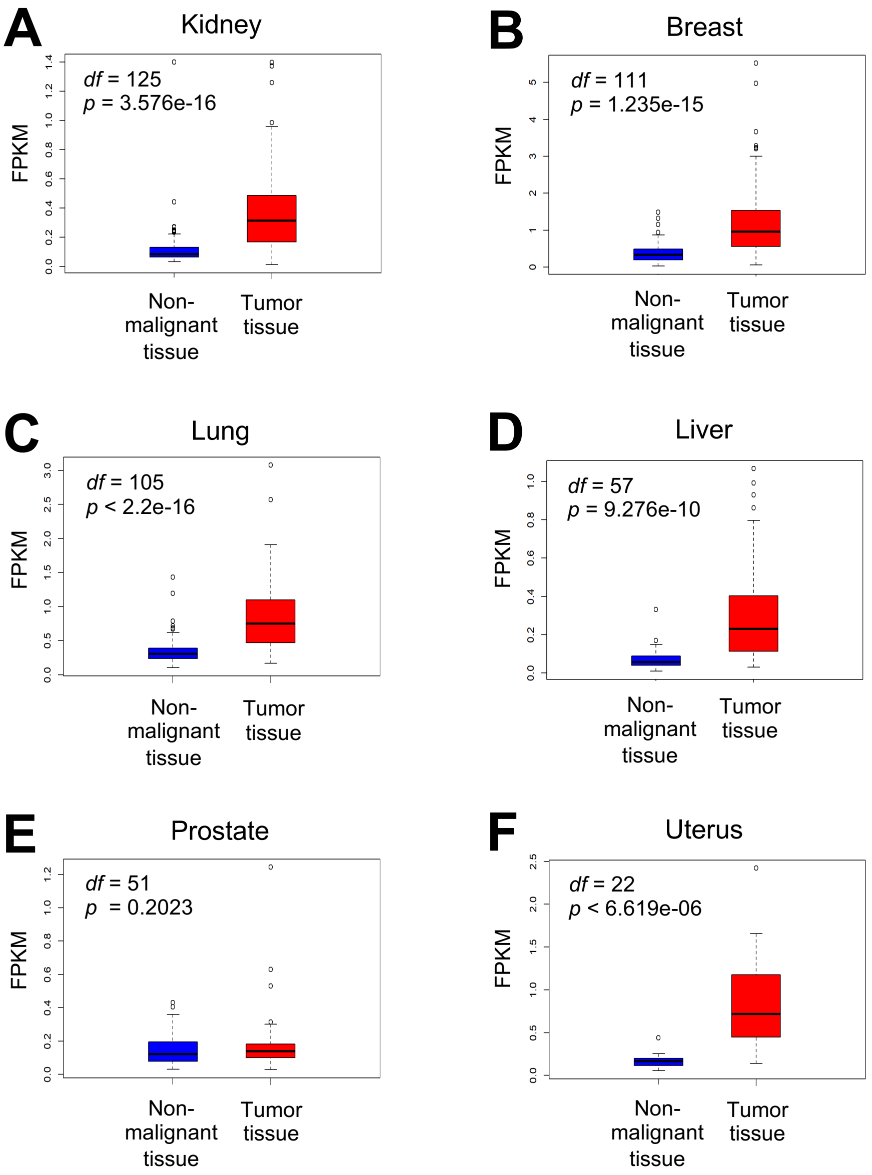

Fig. S7. BRCA2 mRNA expression between tumor tissue and matched non-malignant tissue per individual from 6 different cancer types including kidney (**A**), breast (**B**), lung (**C**), liver (**D**), prostate (**E**), and uterus (**F**).

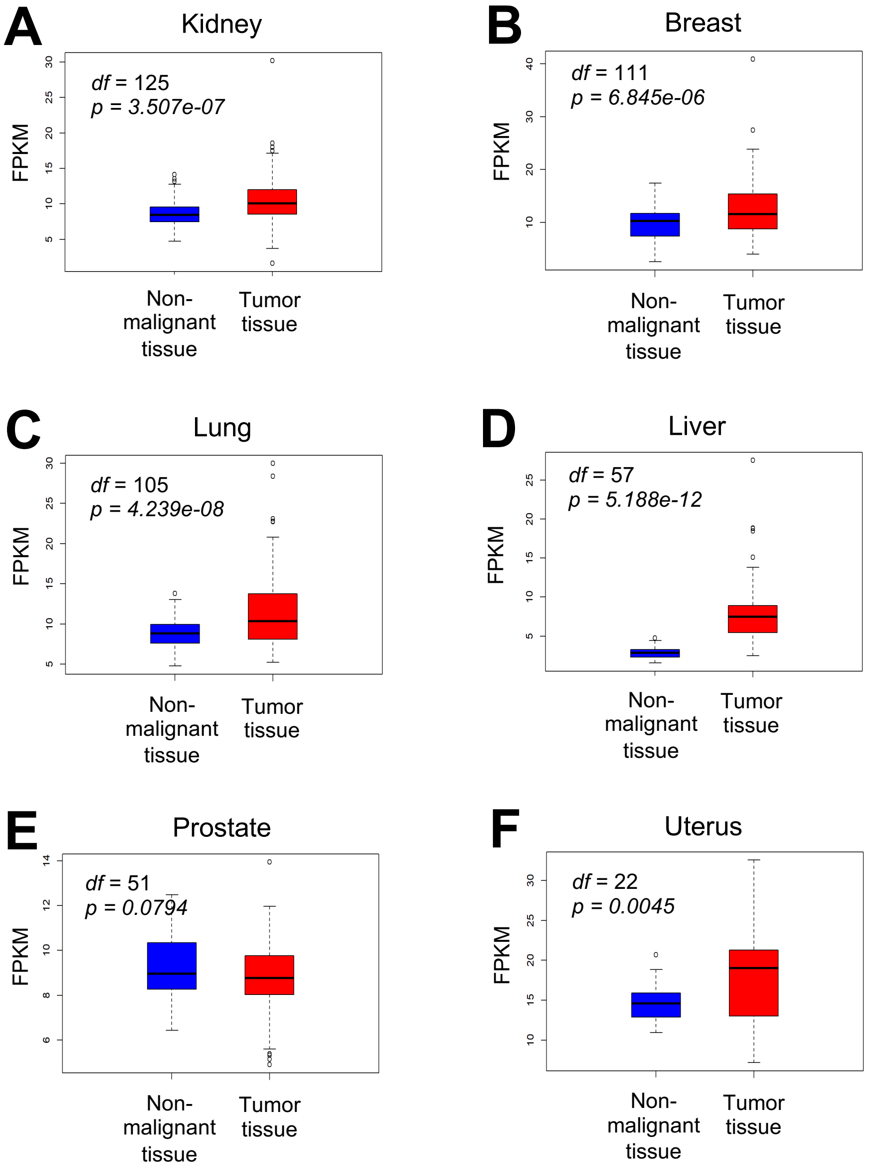

Fig. S8. XRCC1 mRNA expression between tumor tissue and matched non-malignant tissue per individual from 6 different cancer types including kidney (**A**), breast (**B**), lung (**C**), liver (**D**), prostate (**E**), and uterus (**F**).

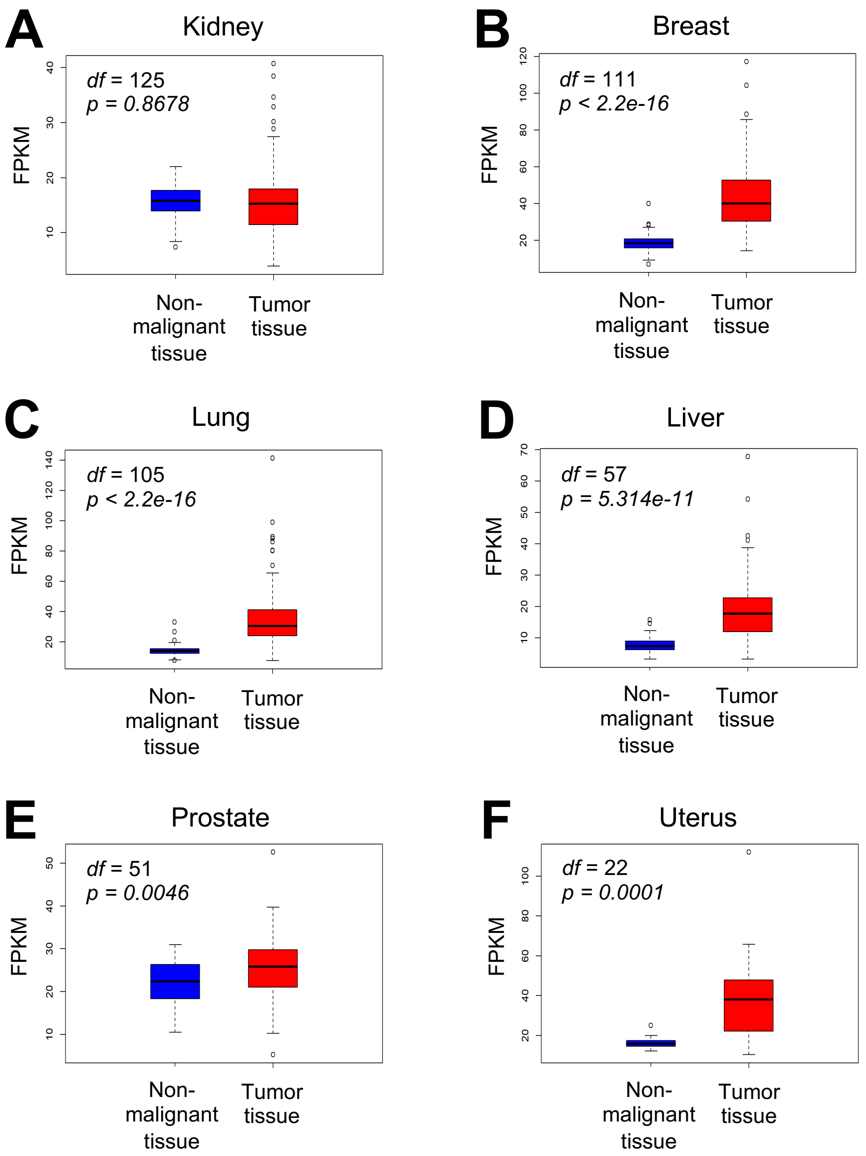

Fig. S9. PARP1 mRNA expression between tumor tissue and matched non-malignant tissue per individual from 6 different cancer types including kidney (**A**), breast (**B**), lung (**C**), liver (**D**), prostate (**E**), and uterus (**F**).

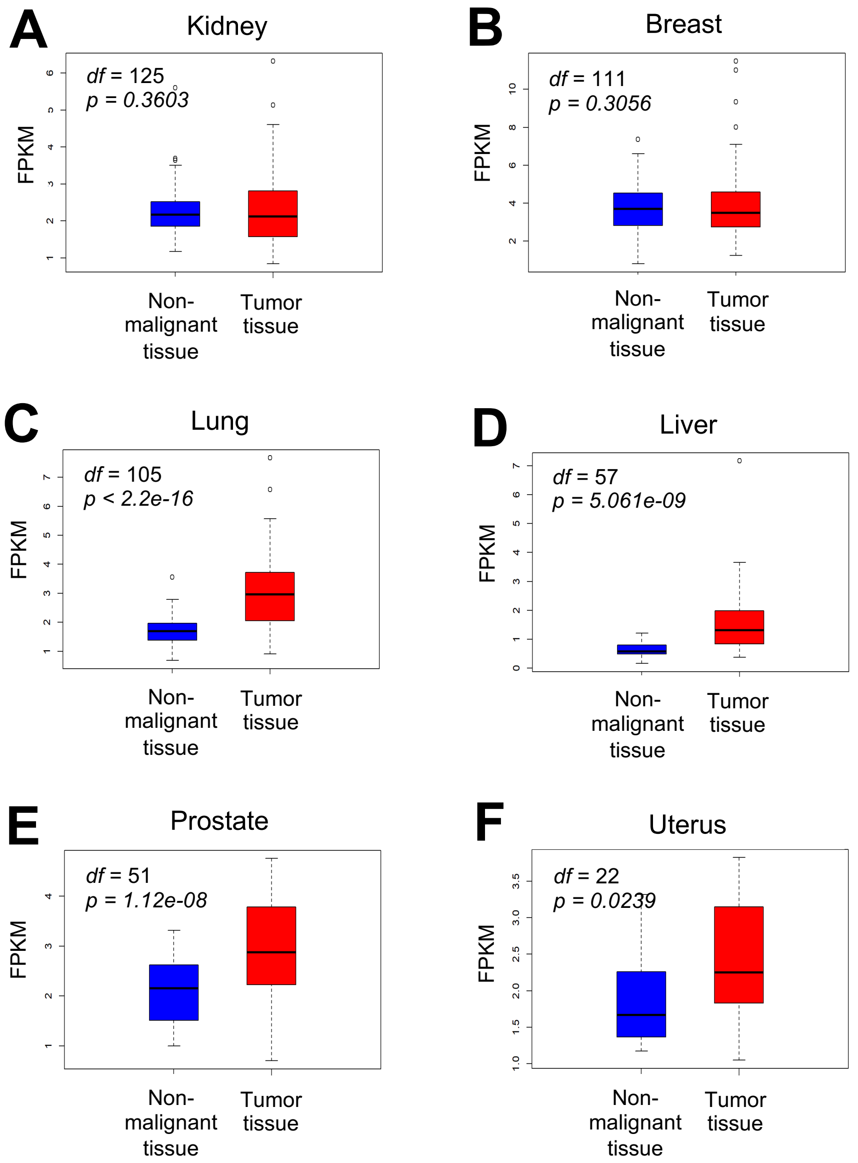

Fig. S10. ATR mRNA expression between tumor tissue and matched non-malignant tissue per individual from 6 different cancer types including kidney (**A**), breast (**B**), lung (**C**), liver (**D**), prostate (**E**), and uterus (**F**).

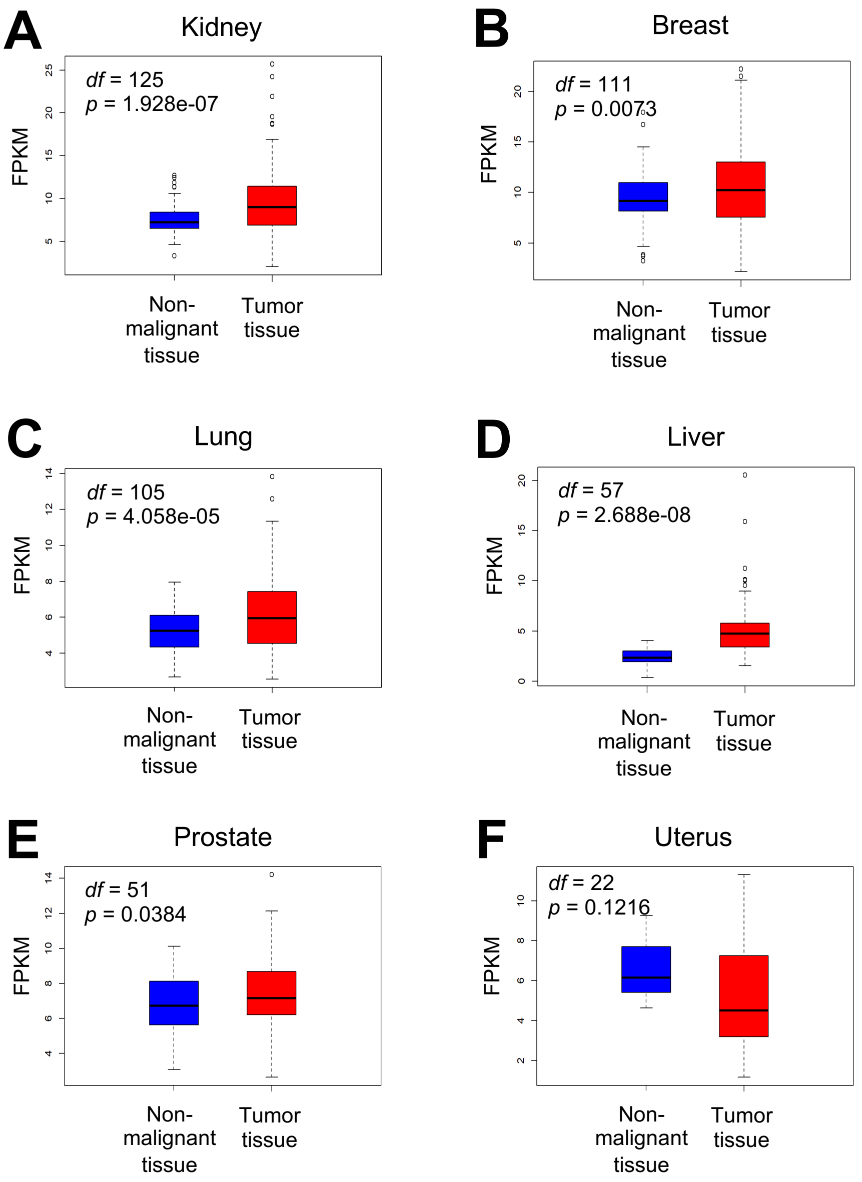

Fig. S11. Rad50 mRNA expression between tumor tissue and matched non-malignant tissue per individual from 6 different cancer types including kidney (**A**), breast (**B**), lung (**C**), liver (**D**), prostate (**E**), and uterus (**F**).

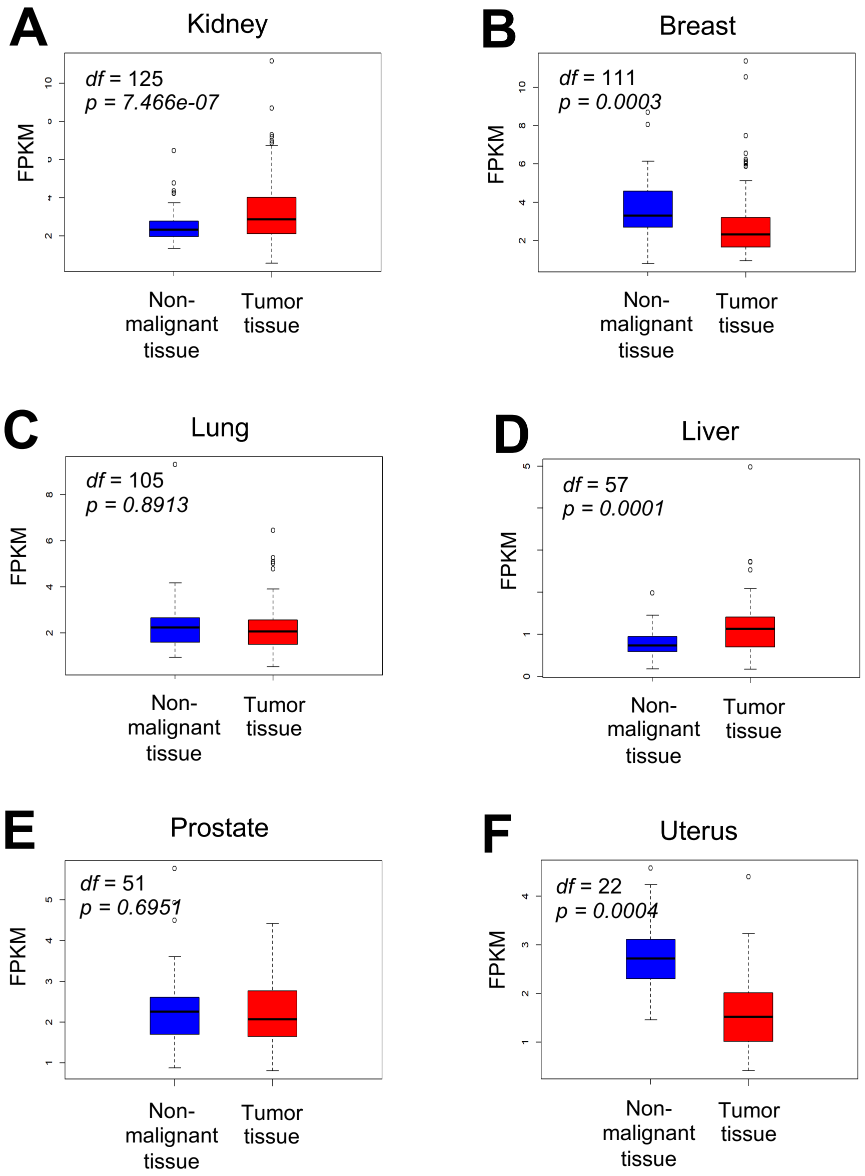

Fig. S12. ATM mRNA expression between tumor tissue and matched non-malignant tissue per individual from 6 different cancer types including kidney (**A**), breast (**B**), lung (**C**), liver (**D**), prostate (**E**), and uterus (**F**).

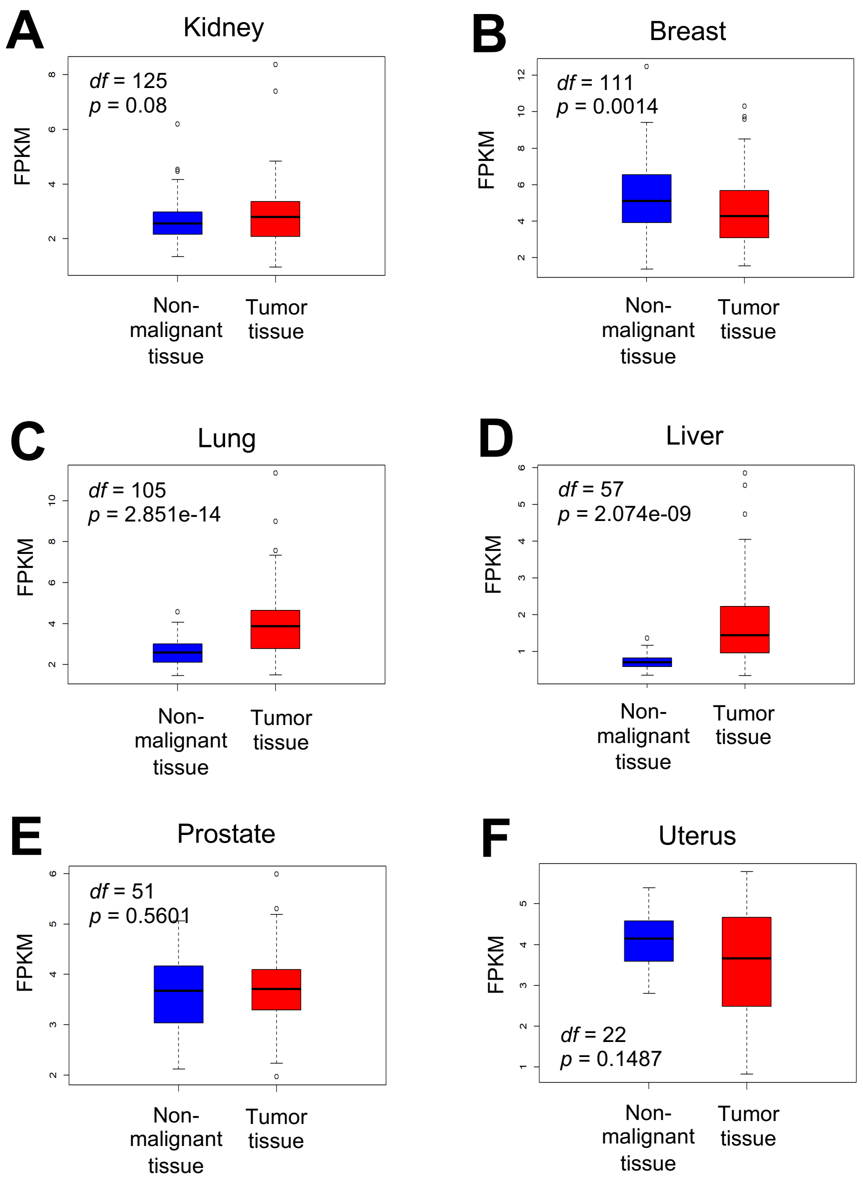

Fig. S13. Mre11 mRNA expression between tumor tissue and matched non-malignant tissue per individual from 6 different cancer types including kidney (**A**), breast (**B**), lung (**C**), liver (**D**), prostate (**E**), and uterus (**F**).

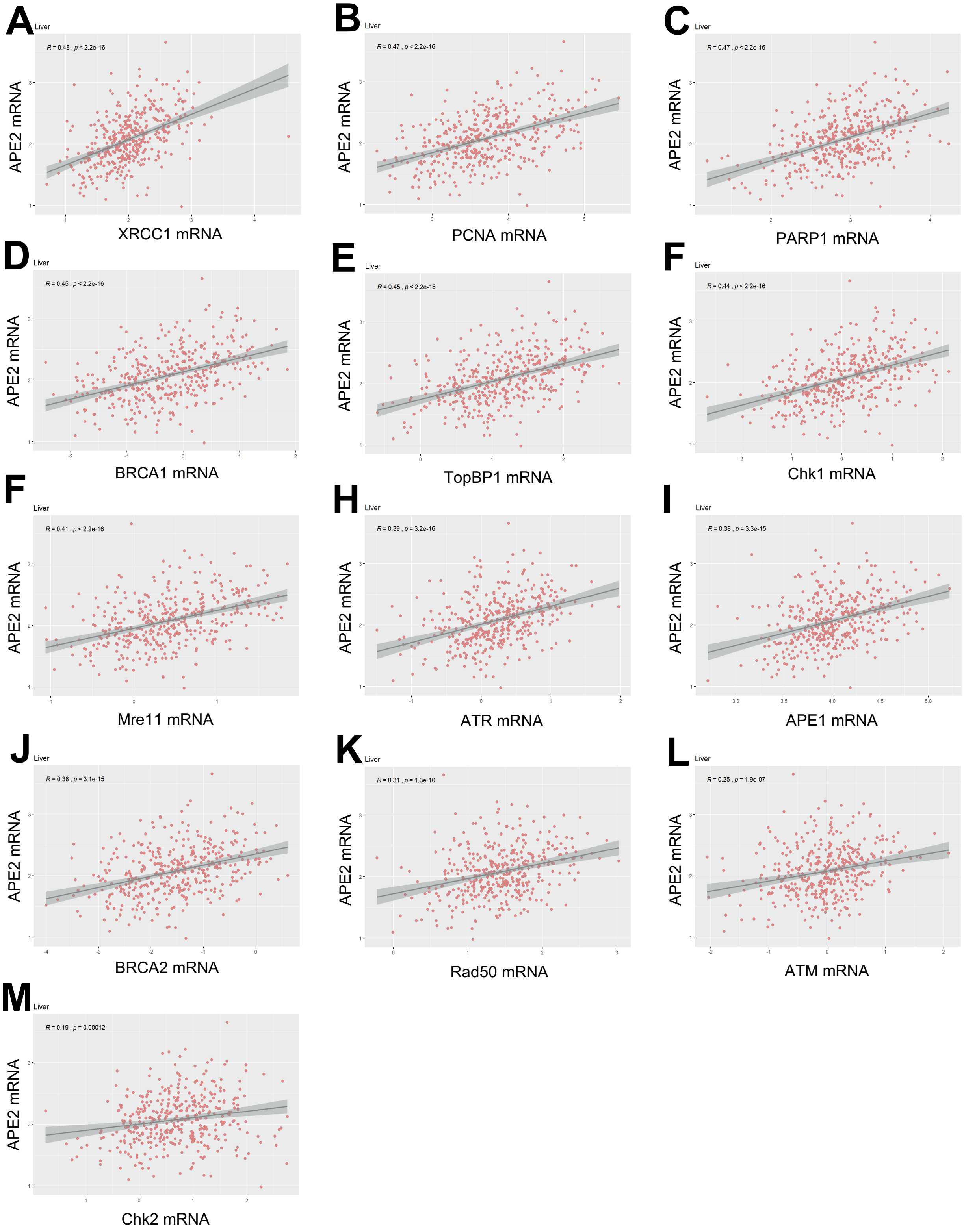

Fig. S14. Correlation between mRNA expression of APE2 and 13 other DNA repair and DDR proteins in tumor tissues of liver cancer. *R* and *p* values are listed in each panel.

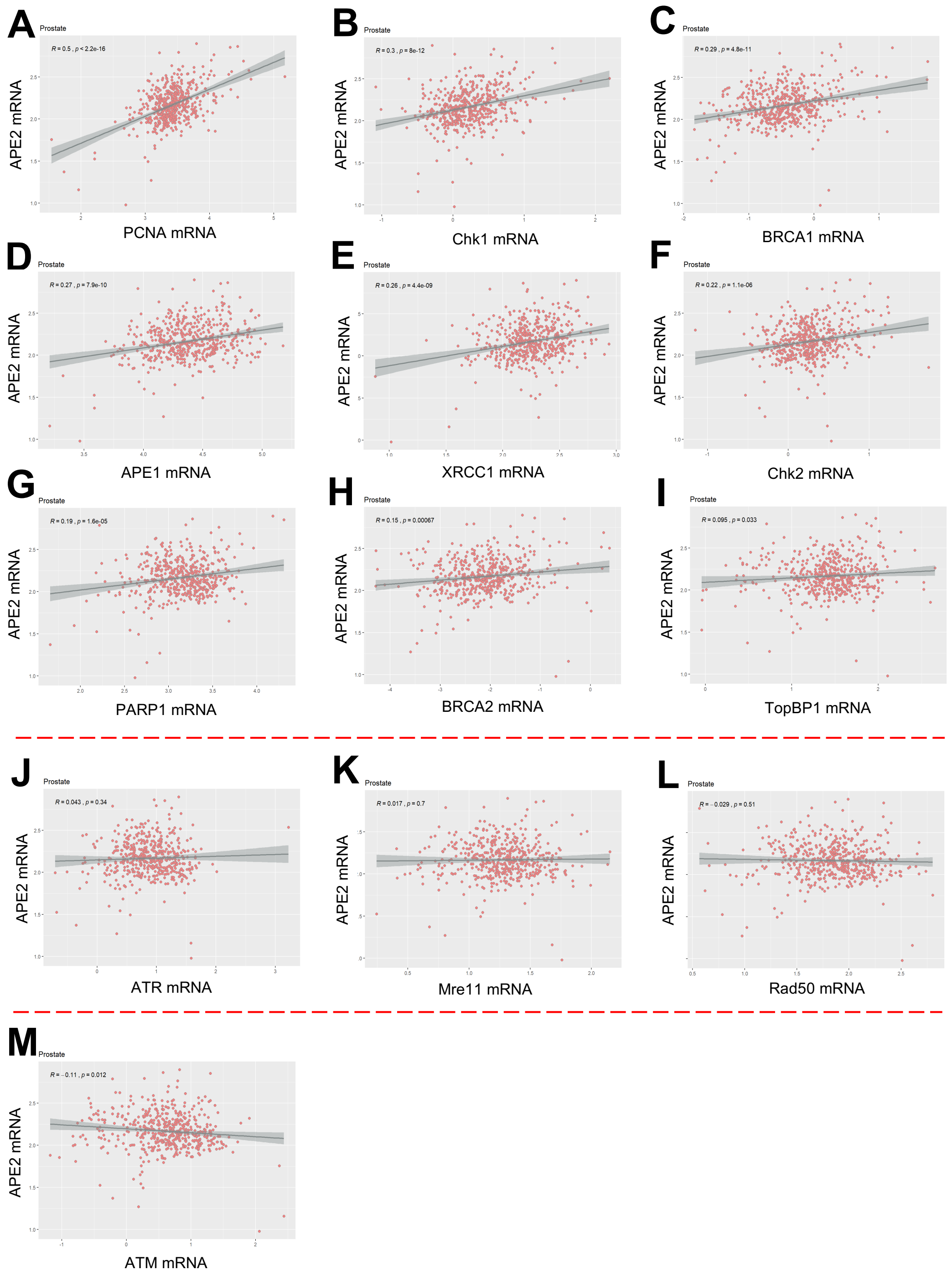

Fig. S15. Correlation between mRNA expression of APE2 and 13 other DNA repair and DDR proteins in tumor tissues of prostate cancer. *R* and *p* values are listed in each panel.

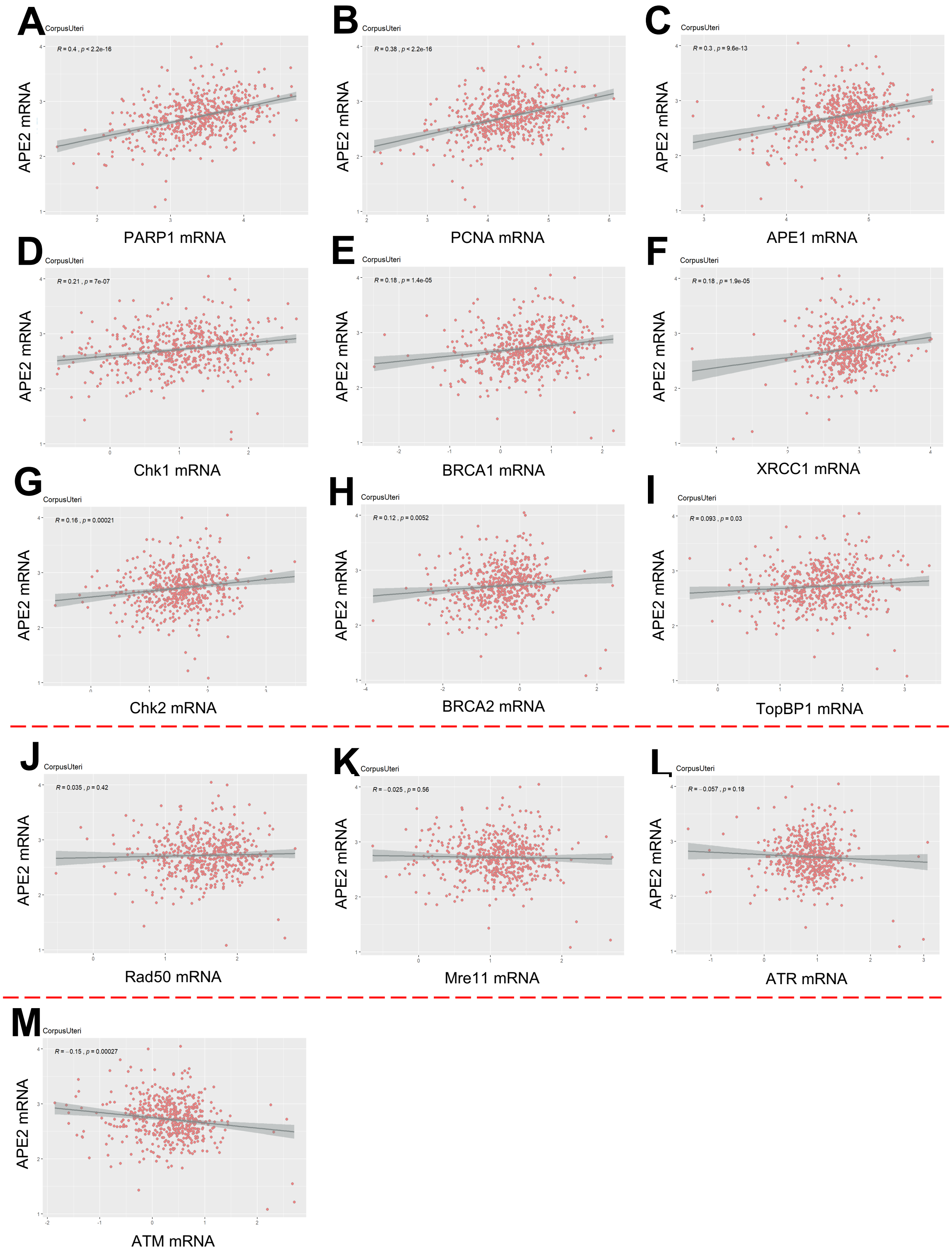

Fig. S16. Correlation between mRNA expression of APE2 and 13 other DNA repair and DDR proteins in tumor tissues of uterine cancer. *R* and *p* values are listed in each panel.

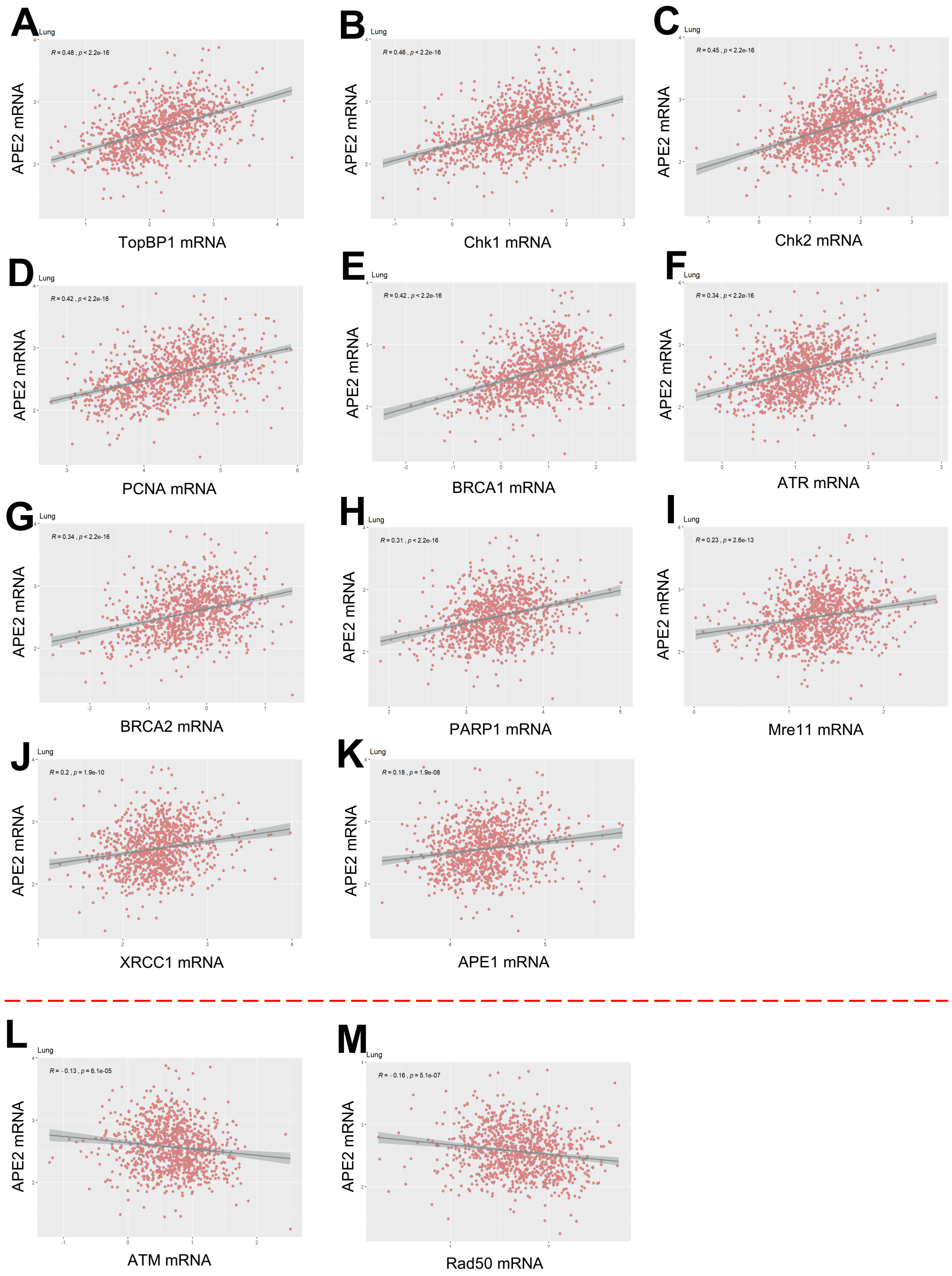

Fig. S17. Correlation between mRNA expression of APE2 and 13 other DNA repair and DDR proteins in tumor tissues of lung cancer. *R* and *p* values are listed in each panel.

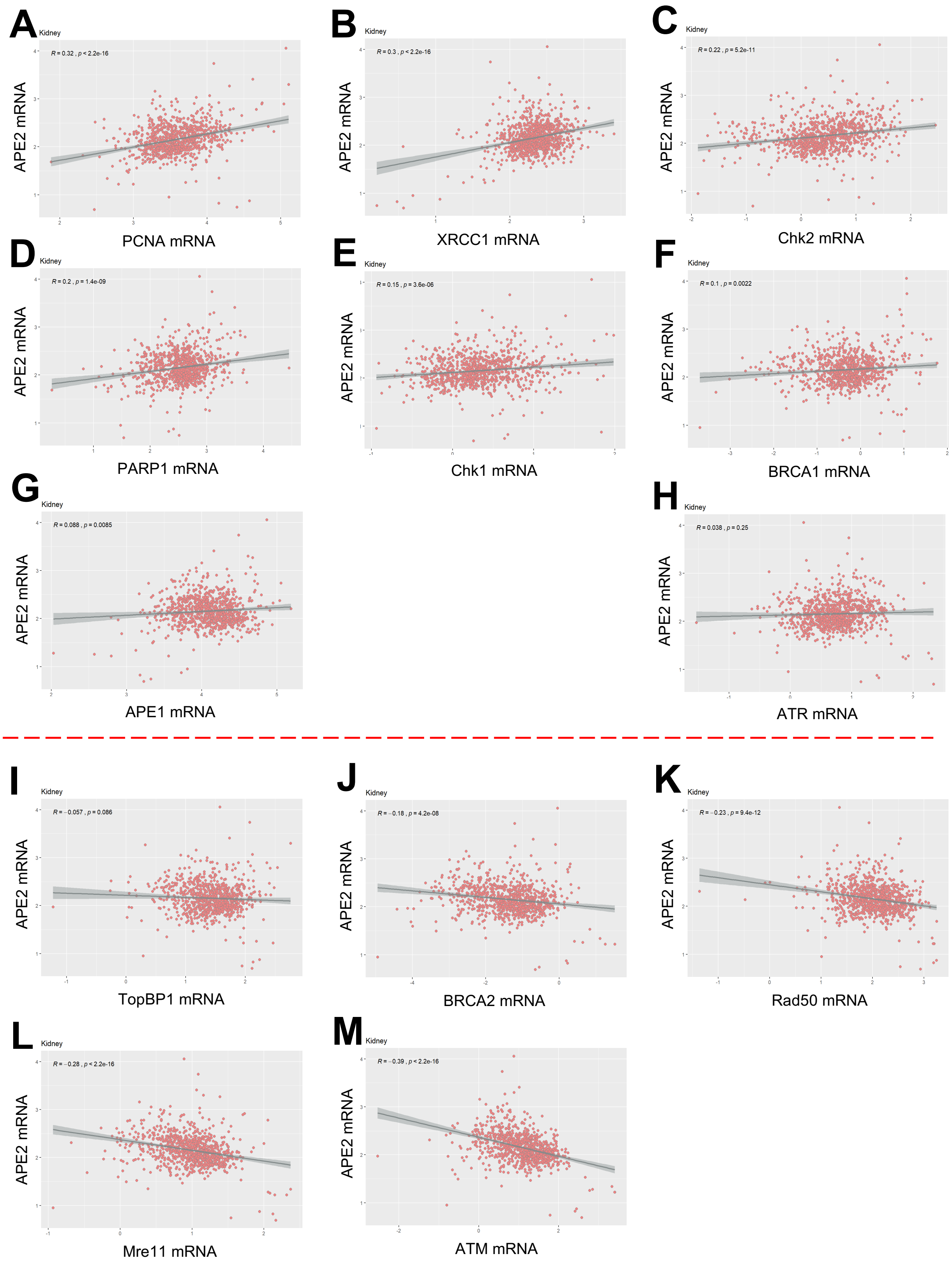

Fig. S18. Correlation between mRNA expression of APE2 and 13 other DNA repair and DDR proteins in tumor tissues of kidney cancer. *R* and *p* values are listed in each panel.
